# Novel Spray Dried Glycerol 2-Phosphate Cross-Linked Chitosan Microparticulate Vaginal Delivery System—Development, Characterization and Cytotoxicity Studies

**DOI:** 10.3390/md14100174

**Published:** 2016-09-28

**Authors:** Emilia Szymańska, Marta Szekalska, Robert Czarnomysy, Zoran Lavrič, Stane Srčič, Wojciech Miltyk, Katarzyna Winnicka

**Affiliations:** 1 Department of Pharmaceutical Technology, Faculty of Pharmacy, Medical University of Białystok, Mickiewicza 2c, Białystok 15-222, Poland; marta.szekalska@umb.edu.pl; 2 Department of Synthesis and Technology of Drug, Faculty of Pharmacy, Medical University of Białystok, Kilińskiego 1, Białystok 15-089, Poland; robert.czarnomysy@umb.edu.pl; 3 Department of Pharmaceutical Technology, Faculty of Pharmacy, University of Ljubljana, Aškerčeva cesta 7, Ljubljana SI-1000, Slovenia; zoran.lavric@ffa.uni-lj.si (Z.L.); stanko.srcic@ffa.uni-lj.si (S.S.); 4 Department of Pharmaceutical Analysis, Faculty of Pharmacy, Medical University of Białystok, Mickiewicza 2d, Białystok 15-222, Poland; wmiltyk@umb.edu.pl

**Keywords:** chitosan, microparticles, glycerol 2-phosphate, spray drying, mucoadhesiveness, cytotoxicity

## Abstract

Chitosan microparticulate delivery systems containing clotrimazole were prepared by a spray drying technique using glycerol 2-phosphate as an ion cross-linker. The impact of a cross-linking ratio on microparticle characteristics was evaluated. Drug-free and drug-loaded unmodified or ion cross-linked chitosan microparticles were examined for the in vitro cytotoxicity in VK2/E6E7 human vaginal epithelial cells. The presence of glycerol 2-phosphate influenced drug loading and encapsulation efficacy in chitosan microparticles. By increasing the cross-linking ratio, the microparticles with lower diameter, moisture content and smoother surface were observed. Mucoadhesive studies displayed that all formulations possessed mucoadhesive properties. The in vitro release profile of clotrimazole was found to alter considerably by changing the glycerol 2-phosphate/chitosan ratio. Results from cytotoxicity studies showed occurrence of apoptotic cells in the presence of chitosan and ion cross-linked chitosan microparticles, followed by a loss of membrane potential suggesting that cell death might go through the mitochondrial apoptotic pathway.

## 1. Introduction

Microparticulate drug delivery systems—multiunit carriers composed of spherical particles with average diameter 1–500 μm—have gained particular interest in the pharmaceutical field, due to their ability to assure prolonged drug release profile, providing stability of labile substances or reducing toxicity of active agents [1,2]. In addition, a large surface area of the microparticles enables assuring more uniform drug absorption, which might be favorable, especially with respect to topical delivery vehicles [3]. In designing microparticulate dosage forms intended for local delivery (buccal, nasal, vaginal, pulmonal), a particularly important aspect is to provide an intimate contact between drug carrier and the mucosal surface. To prolong residence time of the multiunit drug carriers at the site of administration, mucoadhesive polymers are employed [4,5,6]. Among natural polymers, chitosan is one of the most extensively used in technology of microparticle preparation [7,8,9].

Chitosan—a natural multifunctional polysaccharide, comprised of glucosamine and *N*-acetylglucosamine units—is obtained by deacetylation of chitin possessed from exoskeleton of insects, crustaceans or fungi [10,11]. Due to its biocompatibility, biodegradability, intrinsic antimicrobial and penetration enhancement properties, chitosan is considered a useful compound in pharmaceutical technology [12,13,14]. Chitosan’s hydration and gel formation abilities give it the opportunity to prolong release of the drug at the administration site [15]. Additionally, owing to mucoadhesive properties arising from the cationic behavior and the presence of free amine and hydroxyl groups, chitosan is capable of interacting with mucin by electrostatic and hydrogen bonds [16]. Recently, chitosan microparticles have been shown to be useful in oral [17], vaginal [18], nasal [19] and ocular drug delivery [9].

A number of novel techniques have been investigated to develop micro- and nanoparticulate drug carrier preparation, including pressurized gyration [20], microfluidic [21], spray drying [22] and electrohydrodynamic (electrospraying or electrospinning) processing [23,24,25]. Among various encapsulation techniques, spray drying is an advanced, easy to scale-up method of chitosan microparticle preparation, in which dry particles are obtained from a fluid state by evaporating the solvent. Spray drying offers a very flexible control over microparticle properties such as size, flow characteristics and drug encapsulation efficacy [26,27]. This method is uncomplicated, but, in order to receive microparticles with desirable properties, it requires an understanding of the process and careful adjustment of the spray-drying conditions [28].

Despite these advantages, a serious inconvenience of chitosan microparticles is their high solubility in an acidic environment, and, as a consequence, limited ability to control the drug release rate. In order to avoid too rapid dissolution of the polymer formulation and escape of the drug from the microparticle matrix, chemical or physical cross-linking of the chitosan backbone is being practiced [29,30,31]. Glycerol 2-phosphate (β-glycerophosphate disodium, βGP) is a non-toxic divalent ion that interacts with chitosan through electrostatic forces, creating ionic cross-linked networks [32]. It should be noted that in the presence of βGP (used in a proper concentration), chitosan becomes thermally sensitive and may undergo sol/gel transition around body temperature. Recently, chitosan/βGP material has attracted considerable attention as a promising tool for a variety of applications, such as local drug carriers or injectable delivery systems for tissue engineering [33,34]. In addition, data previously published by our group showed that ionic interaction between βGP and chitosan improved mechanical properties of hydrogels and enabled prolonged drug release profile [35,36].

The aim of this work was to design and prepare vaginal mucoadhesive microparticles using chitosan or chitosan/βGP by the spray drying method. Obtained microparticles would be utilized to prepare multiple-unit dosage form intended for topical administration. Particular effort was made to investigate whether the presence of βGP cross-linked chitosan could improve the physicochemical properties of designed microparticulate delivery systems or influence the drug release profile. Clotrimazole—CLO—an imidazole derivative commonly used as the drug of choice for the fungal infections of the urogenital tract—was employed as a model agent. The prepared microparticles were analysed for drug loading, encapsulation efficacy, production yield, surface morphology, hydration capability and the in vitro release. Drug-polymer-cross-linking agent interactions in the solid state were investigated by differential scanning calorimetric analysis. In addition, to examine the mucoadhesive properties of the obtained microparticles, the ex vivo residence time, maximum detachment force and work of adhesion in the presence of porcine vaginal mucosa were evaluated.

Furthermore, this study concentrated on the cytotoxicity examination of microparticles with chitosan or chitosan cross-linked with different ratios of βGP by 3-(4,5-dimethylthiazol-2-yl)-2,5-diphenyltetrazolium bromide MTT assay followed by three independent methods of apoptosis evaluation: fluorescent microscopy, flow cytometry assessment of annexin V conjugated to green-fluorescent FITC dye (annexin V-FITC binding and analysis of mitochondrial membrane potential. The in vitro cytotoxicity profile of both drug-free and drug-loaded microparticles using human vaginal mucosa epithelium VK2/E6E7 was reported for the first time.

## 2. Results and Discussion

### 2.1. Characterization of Microparticles

Chitosan and ion cross-linked chitosan microparticles for vaginal delivery of CLO were prepared for the first time by spray drying method using βGP as a cross-linking agent. Preliminary experiments were performed in order to select suitable amount of excipients and optimal operating conditions of the process. The characteristics of formulated microparticles F1–F6 were summarized in Table 1.

The production yield was in the range from 73.5% ± 0.9% (F3) to 80.6% ± 1.9% (F6) and was found to be higher for βGP/chitosan microparticles. The increase in drug/polymer ratio from 1:4 to 1:3 resulted in a substantial improvement in CLO loading and encapsulation efficacy. The presence of βGP had a negative impact on the drug-loading of microparticles regardless of the amount of chitosan or drug used. Nevertheless, by increasing the ratio of βGP:chitosan (from 1:3 to 1:2), the rise in the encapsulation efficacy was noticed (from 59.6% ± 1.0% to 73.2% ± 9.0%). The moisture’s content was found to be in the range from 6.5% ± 2.4% (formulation F5) to 14.3% ± 3.5% (F1). Microparticles with βGP/chitosan exhibited lower water content compared to non-cross-linked formulations (Table 1).

### 2.2. SEM and DSC Studies

**Figure 1:**
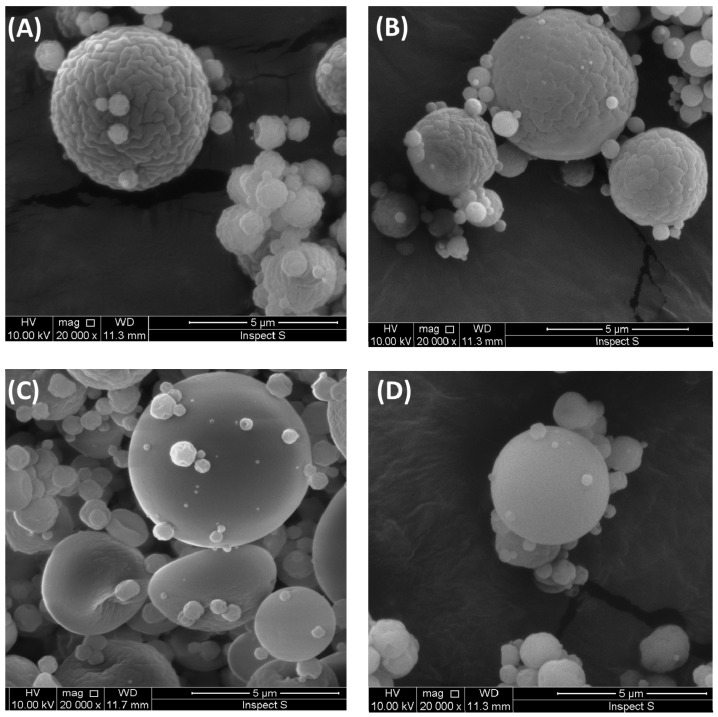
Representative SEM images of clotrimazole (CLO)-loaded microparticles prepared with: (**A**) unmodified chitosan (F3); (**B**) βGP/chitosan 1:3 (*w*/*w*) (F4); (**C**) βGP/chitosan 1:2 (*w*/*w*) (F5); (**D**) βGP/chitosan 1.5:2 (*w*/*w*) (F6); and original magnification ×20,000.

To assess the surface morphology and size of microparticles F3–F6 (with CLO:chitosan ratio 1:3), SEM studies were carried out. The representative SEM photographs of drug-loaded microparticles with non-cross-linked chitosan or βGP/chitosan were presented in Figure 1. In all investigated formulations, the predominance presence of spherical forms was observed. In the case of microparticles F5 and F6 with βGP/chitosan, some elliptical shape particles that resemble biconcave discs in structure were also noticed (Figure 1C). The microparticles F3 with unmodified chitosan were found to be uniform with ridged surface. Notably, with increasing the amount of βGP in chitosan microparticles, the wrinkles on the particles’ surface disappeared and the smoothest and most intact surface in formulations F5 and F6 was observed under identical magnification (×20,000). The diameter range of drug-loaded microparticles was diversified and varied between 0.93 (F6) and 6.33 μm (F4) (Table 1). Interestingly, the mean diameter of βGP/chitosan microparticles decreased from 3.96 ± 2.45 μm (F4) to 2.54 ± 1.59 μm (F6) with increasing βGP cross-linking ratio.

**Figure 2:**
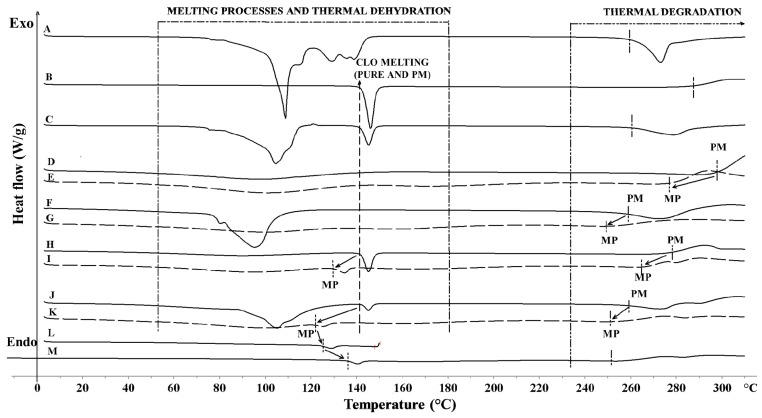
DSC thermograms of: (**A**) βGP powder; (**B**) CLO powder; (**C**) CLO/βGP 1:1 (*w*/*w*) physical mixture (PM); (**D**) chitosan powder; (**E**) placebo microparticles (MP) P1; (**F**) chitosan/βGP 1:1 (*w*/*w*) physical mixture (PM); (**G**) placebo microparticles (MP) P2; (**H**) CLO/chitosan 1:1 (*w*/*w*) physical mixture (PM); (**I**) microparticles F1 (MP); (**J**) CLO/chitosan/βGP (F2) physical mixture (PM); (**K**) microparticles (MP) F2; (**L**) MP F2 heated to 110 °C; (**M**) MP F2 heated for 1 h at 110 °C.

The differential scanning calorimetry DSC thermogram of a CLO sample revealed onset of melting occurring at 142 °C and thermal decomposition starting at about 286 °C (Figure 2). Pure chitosan exhibited a broad endotherm between 10 °C and 140 °C (ΔH 188 J/g) corresponding to its water loss. Chitosan decomposition process occurred around 296 °C [37]. The DSC curve of pure βGP exhibited numerous peaks in the range 80 °C to 150 °C—characteristic for water loss and process of βGP melting [38]—and a decomposition peak at about 264 °C.

The results from DSC studies of physical mixtures (Figure 2C,H,J) displayed no interactions between the drug and chitosan or βGP, whereas a decrease in CLO peak intensity in the thermograms of F1 and F2 microparticles (Figure 2I,K) was noticed indicating a partial loss of drug crystallinity within the polymer matrix. An observed shift to a lower CLO melting temperature (T_melt_) in F1 (130 °C) and F2 microparticles (120 °C) thermal profiles, followed by a decrease in temperature of thermal decomposition (266 and 255 °C for F1 and F2, respectively), might be caused by increasing interactions of CLO with the chitosan matrix and more rigid structure formation. This effect was enhanced with incorporation of βGP cross-linking agent to microparticles. On the contrary, thermal stressing of F2 microparticle samples led to an increase in melting temperature of CLO, which might reflect either alterations in polymorphic forms of the drug or thermal relaxation of chitosan matrix during heating/cooling program cycle. No melting peak of βGP was present in the thermogram of F2 microparticles, which may indicate that βGP was molecularly dispersed within the polymer matrix. The obtained results might suggest that CLO encapsulation in chitosan or chitosan/βGP matrix by using a spray-drying method exerted a stabilizing effect on the drug’s molecular dispersion. The above observations are in agreement with previously reported data in which the preparation method was found to influence the state of the drug in the solid dispersion [39].

### 2.3. Mucoadhesive and in Vitro Drug Release Studies

**Figure 3:**
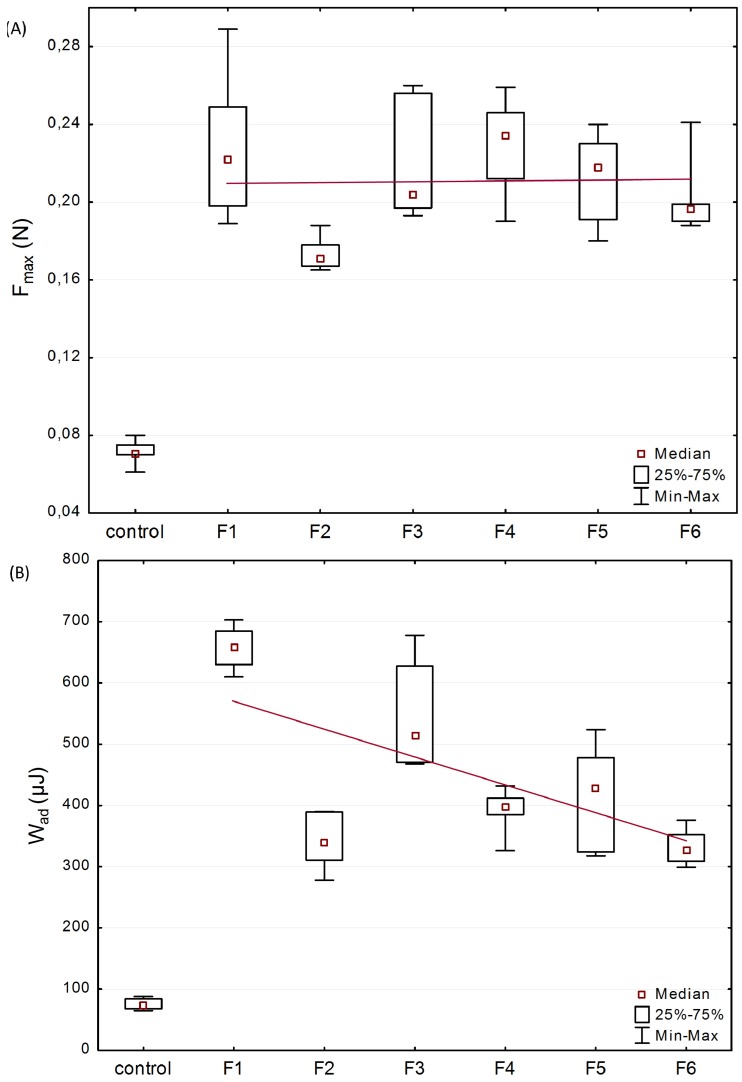
Box-plot graphs presenting mucoadhesive properties: (**A**) maximum force of detachment (*F*_max_); and (**B**) work of adhesion (*W*_ad_) of formulations F1–F6 and control—cellulose paper (median; *n* = 6; trend line plotted through median values).

In order to evaluate microparticles’ characteristics in contact with mucosal surfaces, mucoadhesive studies were accomplished using texture analyser. Porcine vaginal mucosa was applied for the imitation of vaginal mucoadhesion due to its resemblance to the human vagina in terms of anatomical structure, pH, permeability and vaginal secretion [40]. The effect of βGP cross-linking at various ratios, the amount of the drug on the force of detachment (*F*_max_), and work required to overcome the microparticles-porcine vaginal mucosa interaction (*W*_ad_) was presented in Figure 3.

All examined formulations exhibited mucoadhesive properties, expressed as *F*_max_ with the median range from 0.17 N (F2) to 0.23 N (F1 and F4) and *W*_ad_ between 271.8 μJ (F2) and 673.5 μJ (F1). Several studies revealed that the presence of cross-linker reduced chitosan capability of interacting with mucosa as a result of a drop in positively charged free amino groups [41,42]. The preserved ability of βGP/chitosan microparticles to interact with mucosal material could be explained by incomplete chitosan cross-linking with βGP partially retaining protonation of polymers’ functional groups that were still capable of physicochemical interactions with mucosa [43]. Basically, the presence of higher content of CLO crystalline particles in formulations (F1 vs. F3 and F2 vs. F5) did not significantly alter the mucoadhesiveness of the microparticles. Based on the plotted trend line (Figure 3A), it was also noticed that unmodified chitosan and βGP/chitosan microparticles possessed comparable values (*p* > 0.05) of *F*_max_. The obtained data is in agreement with results previously reported by our group, indicating that βGP modification of chitosan in semi-solid drug carriers maintained polymers’ ability to interact with mucosal tissue [35]. Nonetheless, the obtained *W*_ad_ values were found to demonstrate a downward trend (Figure 3B) with raising the amount of ion cross-linker in chitosan microparticles. This drop in work required to separate microparticles mucoadhesive material might be attributed to the presence of a lower amount of water in βGP/chitosan formulations (compared with unmodified chitosan microparticles) responsible for formation of less cohesive structure with mucus. It should also be pointed out that the rough surface of unmodified chitosan microparticles (as shown in Figure 1A) might favor an intimate contact between mucin and polymer compared to more intact βGP/chitosan formulations.

Additionally, in order to investigate microparticles’ behavior in contact with mucosal surface, the residence time to the porcine vaginal mucosa using a self-constructed apparatus was determined [44]. A capability of increased drug residence time after administration in the vaginal cavity appears to be essential in reducing the dosage frequency and improving the patient compliance. It was observed that examined microparticles F3–F6 adhered immediately to the mucosal surface. Notably, the contact time of formulations with βGP/chitosan with vaginal tissue was considerably higher than those obtained for non-cross-linked chitosan microparticles (Table 1). The residence time was found to correlate with βGP concentration and formulation F6, with the highest βGP:chitosan ratio exhibiting the longest period of contact time with mucosal surface. Nevertheless, it should be noted that, throughout the test, a progressive dissolution and surface erosion of chitosan and βGP/chitosan matrix in acidic pH was observed, and, thus, prolonged residence time of βGP/chitosan microparticles could be attributed to a slower matrix dissolution rate compared to unmodified chitosan formulation.

**Figure 4:**
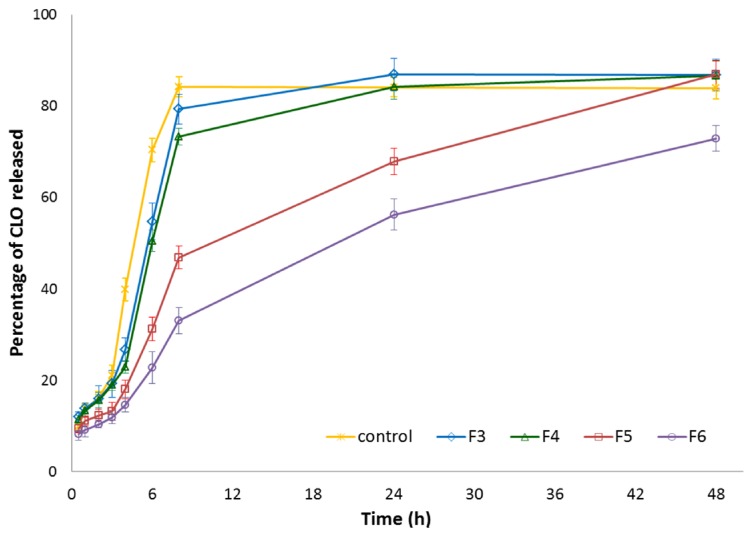
Percentage of CLO released from microparticles with unmodified chitosan (F3) or βGP/chitosan (F4–F6) compared to 1.0% (*w*/*v*) CLO dispersion in the release medium (control) (mean ± SD; *n* = 3).

To examine the influence of βGP cross-linking on CLO release profiles from chitosan microparticles (with CLO:chitosan ratio 1:3), formulation F3 (with unmodified chitosan) and formulations F4–F6 (with different βGP:chitosan ratios) were selected for the in vitro release study. The dissolution test was carried out with using the enhancer cell equipped with Cuprophan membrane. This model reflects the conditions of the vaginal cavity more suitably as it enables maintaining microparticles in a reservoir where drug carriers slowly hydrate and form gel matrix from which drug is released [45]. As shown in Figure 4, all designed chitosan microparticles exhibited prolonged CLO release rates as compared to control—simple dispersion of CLO in the release medium. However, it was found that the drug was certainly faster released from formulation F3 with non-cross-linked chitosan (the plateau was reached within 24 h) as a result of a high degree of protonation of amino groups responsible for electrostatic repulsion between charged chains and expansion of the polymer network. As expected, the amount of ion cross-linker had a remarkable effect on the CLO release from βGP/chitosan microparticles. It was noticed that release behavior from formulation F4 (with 1:3 βGP:chitosan ratio) was comparable (*p* > 0.05) with the data obtained for unmodified chitosan formulation F3, whereas microparticles F5 and F6 (with βGP:chitosan in a ratio 1:2 and 1.5:2 (*w*/*w*), respectively) retarded the release of CLO markedly. The observed phenomenon might be attributed to a more intact structure of microparticles F5 and F6, as indicated by the SEM picture (Figure 1C,D).

**Figure 5:**
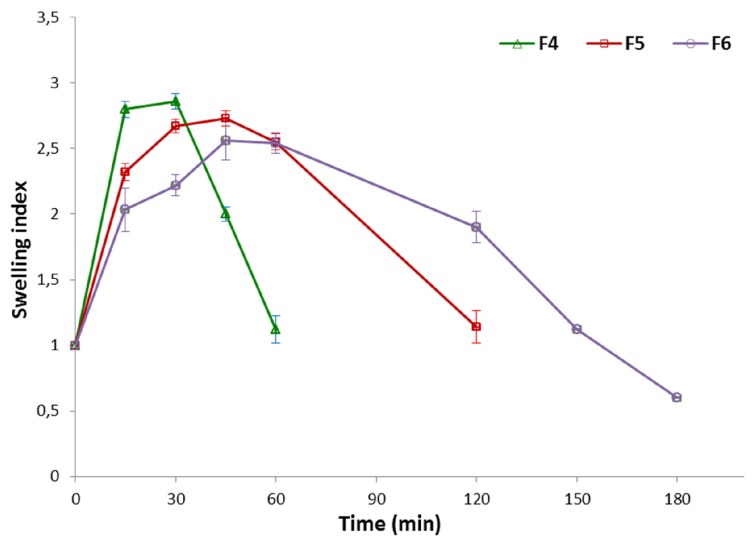
Swelling index study of microparticles F4–F6 (mean ± SD; *n* = 3).

Due to the fact that the drug release behavior from chitosan microparticles is strongly associated with their water uptake properties, swelling index studies of formulations F3–F6 were additionally carried out and reported in Figure 5. As expected, all examined formulations displayed hydration capability at acidic pH resulting from protonation of chitosan amino groups and repulsion of the polymer chain. Microparticles with unmodified chitosan (F3) were found to swell rapidly and dissolve almost immediately after direct contact with acidic buffer solution; thus, it was impossible to measure their accurate degree of swelling. It was noticed that the swelling capacity of formulations with βGP/chitosan decreased with increasing a degree of cross-linking. Furthermore, βGP:chitosan ratio 1:2 (F5) and 1.5:2 (*w*/*w*) (F6) appeared to be essential for preserving microparticles integrity in acidic environment up to 2 and 3 h, respectively. Higher amounts of βGP might have interfered with the process of chitosan chain relaxation, thus leading to a suppression of the relaxation mechanism. As a result, slower disintegration of βGP/chitosan formulations and drug dissolution rate in acidic pH was observed.

### 2.4. In Vitro Cytotoxicity Studies

Chitosan has been widely used for the preparation of microparticulate drug delivery systems. The biocompatibility and non-toxicity of chitosan material was proved in vitro on several types of cell lines and in vivo after intravenous or long-term oral administration [46,47,48]. Despite the fact that chitosan is extensively studied as an excipient in microparticulate dosage forms [5,19,42], its safety profile has still not been fully determined. The polymer biocompatibility may be influenced by the applied processing method or the presence of an active agent. In addition, because of unique physicochemical properties emerging throughout chitosan microparticle preparation (e.g., large surface area), its toxicity profile could remarkably differ in comparison to native polymer material [49]. Therefore, the safety profile of drug-free and CLO-loaded chitosan microparticles prepared by spray drying in VK2/E6E7 cells was evaluated in the present work. As any modification of chitosan structure should be followed by an exhaustive evaluation of its cytotoxicity, a particular effort was made to investigate whether the presence of βGP cross-linker in chitosan microparticles impacted the VK2/E6E7 cell viability. Formulations with unmodified chitosan (placebo P3, drug-loaded F3) and βGP cross-linked chitosan at two different ratios 1:2 (P5, F5) and 1.5:2 (P6, F6) were selected for the cytotoxicity investigations. To exclude the influence of ion cross-linker itself and the pH of the medium on the cell viability, several controls were employed concomitantly with experimental samples and summarized in Table 2.

**Figure 6:**
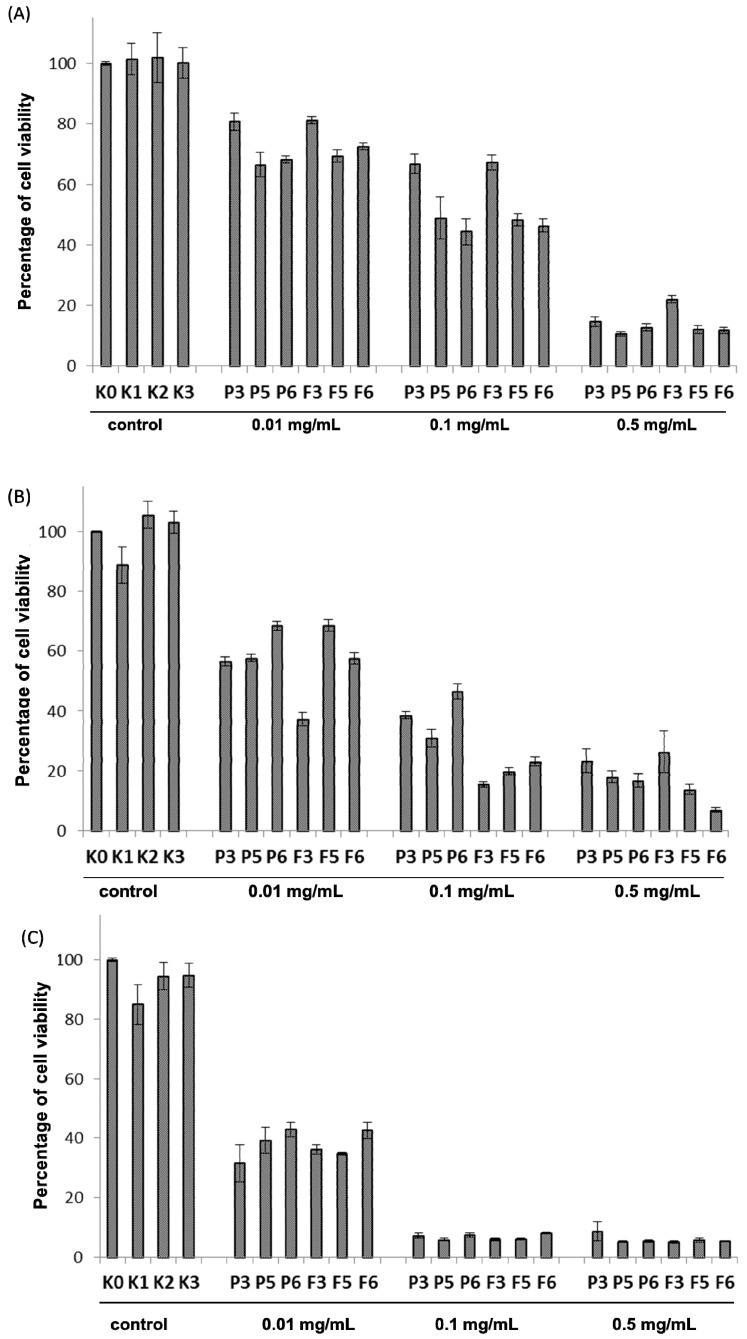
Column charts presenting the viability of VK2/E6E7 cells with controls (as described in Table 2) and different concentrations (expressed in mg/mL) of drug-free (P3, P5, P6) or CLO-loaded (F3, F5, F6) microparticles with unmodified (P3, F3) or chitosan cross-linked with βGP at different ratios (P5–P6, F5–F6) incubated for: (**A**) 4 h; (**B**) 24 h; and (**C**) 48 h measured by using MTT assay (*n* = 6).

The MTT assay, a quantitative and quick colorimetric test was chosen to preliminary screen the cytotoxicity range of drug-free and CLO-loaded chitosan and βGP/chitosan microparticles. All investigated formulations showed dose-dependent and time-dependent cytotoxicity in the concentration range of 0.01 to 0.5 mg/mL as presented in Figure 6. At the lowest applied concentration of chitosan 0.01 mg/mL, microparticles were found to exert low cytotoxicity after 4 h incubation (the range of median of cell viability 66.5%–81.2%), whereas a substantial loss of viable cells (up to median range 31.6%–41.8%) (*p* < 0.05) after 48 h incubation was noticed. Cells exposed to chitosan and βGP/chitosan formulations at concentration 0.5 mg/mL exhibited high cytotoxic effects (median cell viability below 25%) regardless of the incubation time. In addition, a slightly lower inhibitory effect of drug-free microparticles on VK2/E6E7 cell viability compared to CLO-loaded formulations after 24 h incubation was demonstrated. As shown in Figure 6A, βGP/chitosan microparticles caused greater reduction in VK2/E6E7 cells viability (median 66.5%, 68.3% for P5, P6 and 69.4%, 72.5% for F5 and F6, respectively) compared to microparticles with unmodified chitosan (median 80.8% (P3) and 81.2% (F3)) within 4 h incubation, suggesting that ion cross-linked chitosan microparticles exerted stronger impact on the VK2/E6E7 metabolic activity at early time points. Nevertheless, the differences in MTT cytotoxicity between non-cross-linked and ion cross-linked microparticles after 48 h incubation were found to be statistically irrelevant (*p* > 0.05) (Figure 6C).

**Figure 7:**
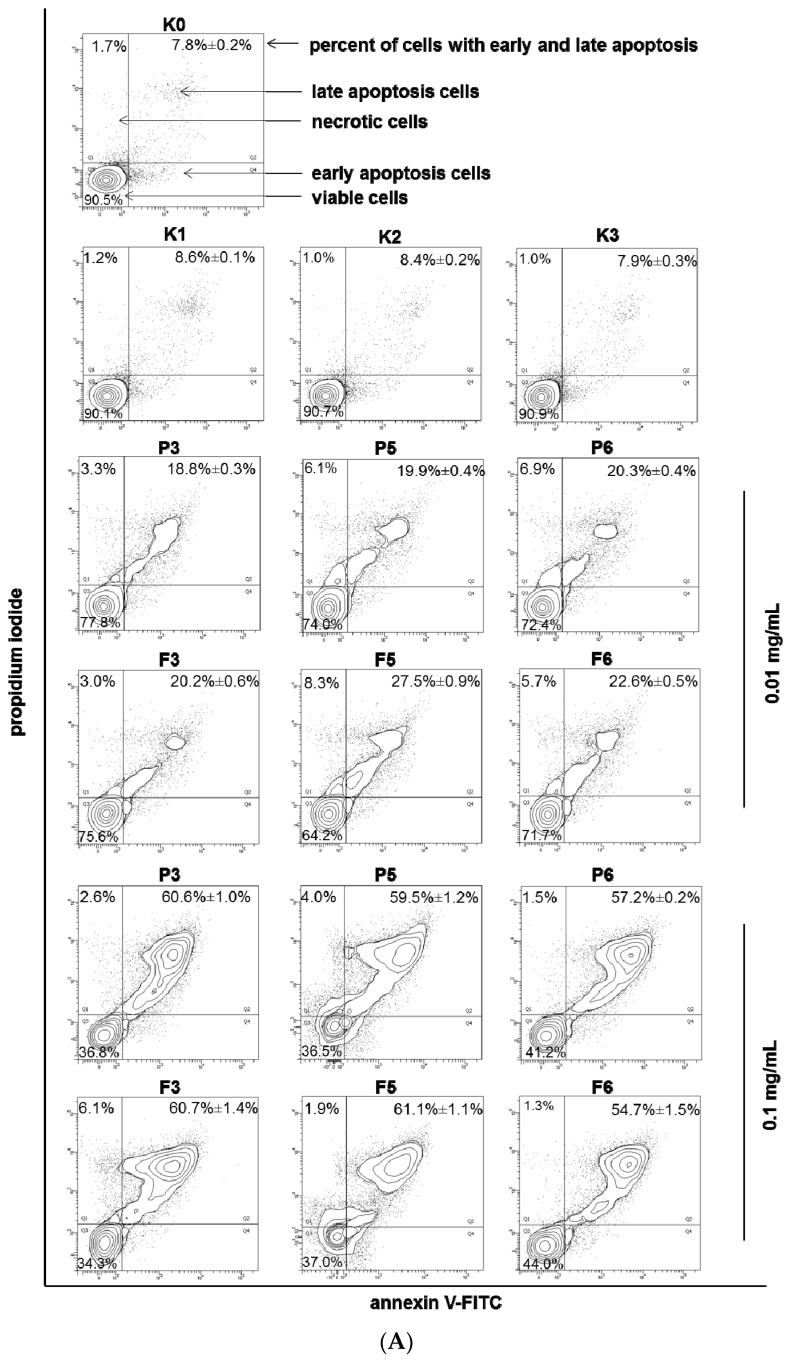

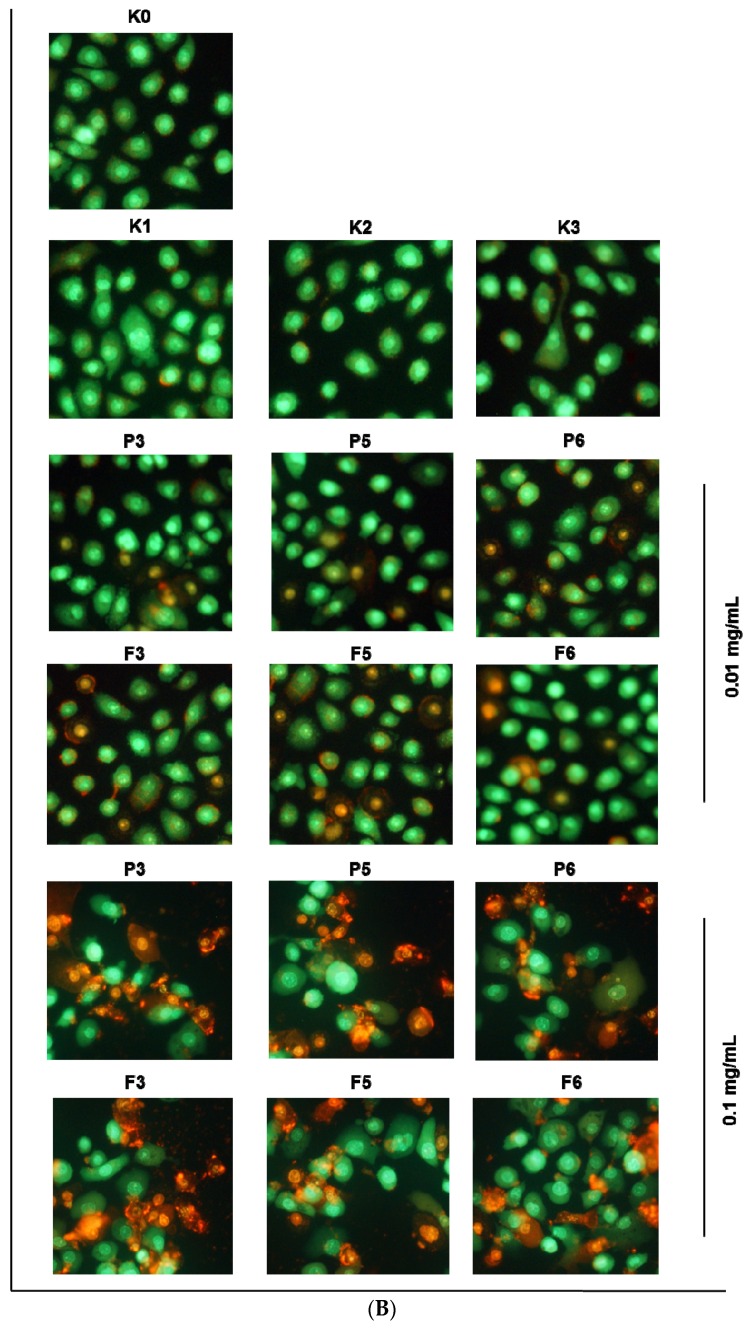
Representative (**A**) flow cytometry dot plots for Annexin V-FITC assay (mean ± SD; *n* = 3); (**B**) fluorescence microscopy images (magnification ×200) of VK2/E6E7 cells incubated with 0.01 or 0.1 mg/mL of drug-free (P) or CLO-loaded (F) chitosan (P3, F3) and βGP/chitosan microparticles (P5–P6, F5–F6) and controls (as described in Table 2) for 24 h.

The MTT assay reflects the metabolic activity of the cells that may be not directly related to cell death as an outcome; therefore, to elucidate the nature of cell death induced by chitosan and βGP/chitosan microparticles in VK2/E6E7 cells, flow cytometric analysis after annexin V-FITC and propidium iodide staining was performed. Based on the preliminary results from the MTT assessment, two microparticle concentrations (which corresponded to chitosan concentration)—0.01 and 0.1 mg/mL—were applied in these studies. The incubation of VK2/E6E7 cells with chitosan and βGP/chitosan microparticles induced visible phosphatidylserine exposure after 24 h of incubation (Figure 7A).

At a concentration of 0.1 mg/mL, the number of early and late apoptotic cells was significantly higher (*p* < 0.05) than at a concentration of 0.01 mg/mL. The differences in the nature of cell death evoked by drug-free and drug-loaded microparticles were found to be statistically insignificant (*p* > 0.05), which suggest that the inhibition of the cell viability is not influenced by the presence of the drug. In addition, the obtained results demonstrated comparable viability of VK2/E6E7 cells within exposures to unmodified and βGP cross-linked chitosan microparticles. Interestingly, the above findings did not correspond with the cytotoxicity studies previously reported by our group in which lesser degrees of cytotoxicity had been noticed in VK2/E6E7 incubated with solutions of chitosan cross-linked with βGP (prepared by simple dissolution of the polymer in acetate buffer without further processing) [35], which might indicate that the applied spray drying process could have influenced the cytotoxicity profile of βGP/chitosan.

VK2/E6E7 cells were also subjected to chitosan and βGP/chitosan microparticle incubation followed by acridine orange-ethidium bromide double staining. Acridine orange—a nucleic acids-selective fluorescent dye—binds to DNA, staining it green, and interacts with RNA, making it appear orange-red. Ethidium bromide is solely absorbed by nonviable cells and emits orange fluorescence by intercalation to chromatin of necrotic cells [50]. The mixture of both dyes is commonly used to detect specific features of apoptosis: apoptotic bodies formation and nuclear envelope disruption. Representative fluorescence microscopy images presented in Figure 7B clearly displayed morphological changes after 24 h incubation with 0.01 and 0.1 mg/mL chitosan and βGP/chitosan microparticles. The results of flow cytometry and fluorescence microscopy assessments revealed that chitosan and βGP/chitosan microparticles induced the VK2/E6E7 cell death via an apoptotic mode.

The cytotoxicity induced by chitosan microparticulate delivery systems is not entirely unexpected as Prego et al. [51] reported a cytotoxic effect of chitosan nanocapsules prepared by the solvent displacement method on human epithelial colorectal adenocarcinoma cells. Investigations performed on tripolyphosphate cross-linked chitosan microparticles obtained by ionotropic gelation also revealed a cytotoxic effect of the polymer on retinal cells in vitro in a dose-dependent manner [9]. In contrast, Pai et al. [52] found that rifampicin and rifabutin-loaded chitosan microparticles (prepared by combination of ionotropic gelation and spray drying technique) exerted no toxic effect in vivo on Sprague-Dawley rats. In addition, the in vitro cytotoxicity of tenofovir-loaded, unmodified and thiolated chitosan nanoparticles confirmed no cytotoxicity in vaginal epithelial cells [53].

**Figure 8:**
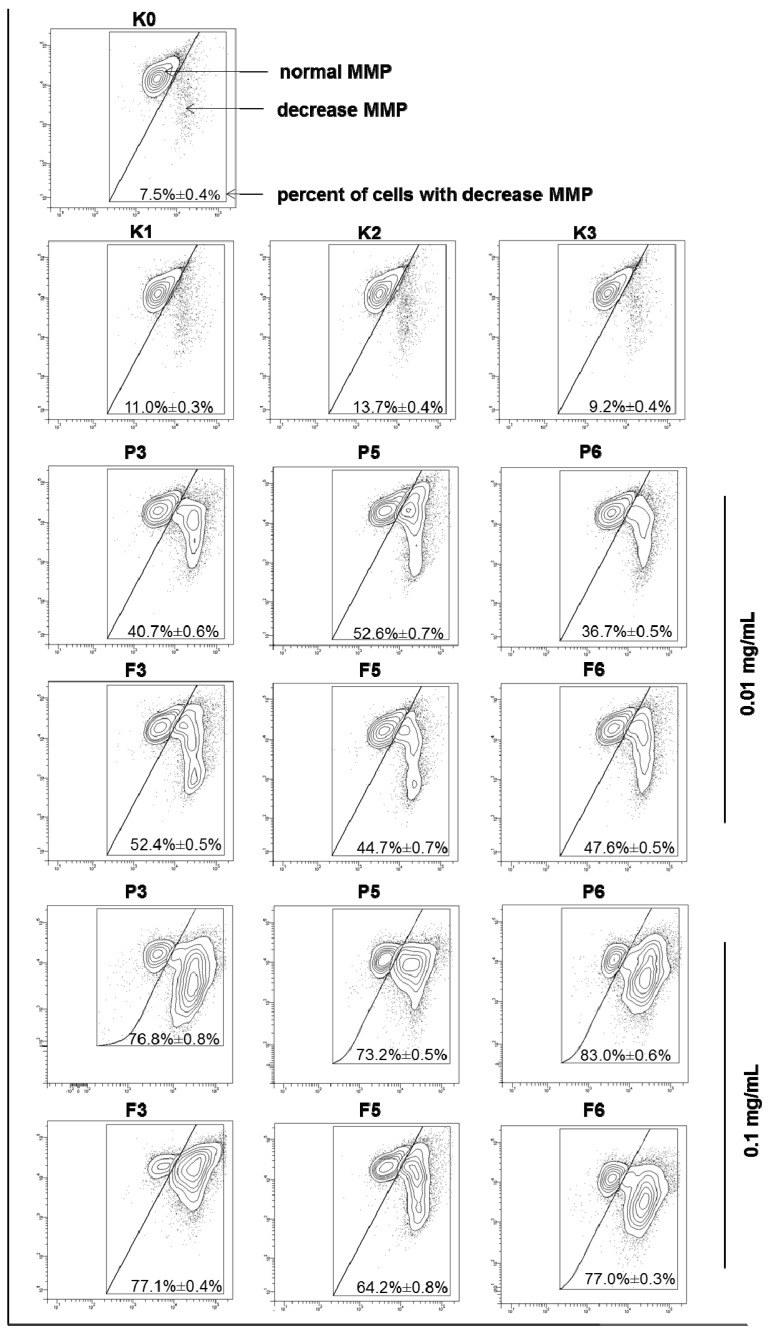
Representative dot-plots presenting the loss of mitochondrial membrane potential (MMP) of VK2/E6E7 cells after 24 h incubation with 0.01 or 0.1 mg/mL of drug-free (P) or CLO-loaded (F) chitosan (P3, F3) and βGP/chitosan microparticles (P5–P6, F5–F6) and controls (as described in Table 2) measured by JC-1 fluorescence (mean ± SD; *n = 3*).

Cytotoxicity was additionally evaluated by the fluorescent mitochondrial probe JC-1—a marker of mitochondrial membrane integrity. Permealization of mitochondrial membrane followed by a decrease in its potential (MMP) is a very useful marker to evaluate induction of apoptosis [54]. In a normal cell, JC-1 is present as a monomer in cytosol (where emits green fluorescence) and as aggregates in mitochondria (emitting red fluorescence), whereas in apoptotic cell with disrupted mitochondrial membrane, the dye retains its monomeric form in mitochondria and produces green fluorescence only. The effects of chitosan and βGP/chitosan microparticles on the MMP of VK2/E6E7 cells after 24 h incubation were displayed in Figure 8.

The percentage of cells with the loss of MMP rose significantly with an increase in chitosan microparticles concentration from 0.01 to 0.1 mg/mL. The JC-1 assay confirmed that mitochondrial functions did not vary markedly (*p* > 0.05) between cells exposed to unmodified chitosan and βGP/chitosan microparticles. The observed mitochondrial membrane disruption in the presence of chitosan microparticulate delivery systems could be explained by the electrostatic interactions of a polymer’s positively charged functional groups in the polymer backbone (partially maintained also in case of βGP/chitosan microparticles as a consequence of incomplete chitosan cross-linking with βGP) with membrane phospholipids. These results are in accordance with those obtained in the annexinV-FITC/PI assessment, pointing out that the apoptosis evoked by chitosan and βGP/chitosan microparticles might go via the mitochondrial pathway.

## 3. Materials and Methods

### 3.1. Materials

High quality chitosan (Chitoscience^®^ product line) with an individual certificate of analysis was obtained from Heppe Medical Chitosan GmbH (Haale, Germany). The number of average molecular weight (80 kDa) and weight average molecular weight (232 kDa) were assessed by Agilent 1260 Infinity GPC/SEC at 35 °C with a refractive index detector (Agilent Technologies, Santa Clara, CA, USA) and PSS Novema columns (PSS Standards Polymer Service GmBh, Mainz, Germany). A sample of 2 mg of chitosan was diluted in 0.3 M formic acid (1 mL) overnight prior analysis. The average viscosity 32 ± 3 mPa·s of 1.0% chitosan in 1.0% acetic acid (*w*/*v*) was measured at 25 °C by Haake Viscotester (Thermo Scientific, Darmstadt, Germany), whereas the moisture content 6.5% ± 0.2% was evaluated by using a moisture analyser Radwag WPS 50SX (Radom, Poland). The deacetylation degree 79.9% was determined by titration method according to Czechowska-Biskup et al. [55].

Clotrimazole was a gift from Ziaja Ltd. (Gdańsk, Poland). Glycerol 2-phosphate disodium salt hydrate, dimethyl sulfoxide (DMSO, anhydrous), Cremophor EL, penicillin, streptomycin, 3-(4,5-dimethylthiazol-2-yl)-2,5-diphenyltetrazolium bromide (MTT), acridine orange, and ethidium bromide were purchased from Sigma Aldrich (Steinheim, Germany). FITC Annexin V Apoptosis Detection Kit II and JC-1 MitoScreen Kit were products of BD Biosciences (San Jose, CA, USA). Porcine vaginal mucosa from white pigs weighing approximately 200 kg was obtained from the veterinary service of an indigenous slaughterhouse (Turośń Kościelna, Poland) and prepared according to Squier et al. [56]. Normal human vaginal epithelial cells (VK2/E6E7; CRL 2616) were obtained from the American Type Culture Collection (Manassas, VA, USA). Keratinocyte serum-free medium (ker-SFM) with a supplement kit was from Life Technologies Sp. z o.o. (Warsaw, Poland). Methanol (HPLC grade) was obtained from Merck (Darmstadt, Germany). Water for HPLC was distilled and passed through a reverse osmosis system Milli-Q Reagent Water System (Billerica, MA, USA). Acetic buffer (pH 4.5) for mucoadhesive, swelling and in vitro release studies was prepared according to Ph. Eur. 8.0 [57] with the following composition (per liter of distilled water): anhydrous sodium acetate 63 g and acetic acid 90 mL; ionic strength (0.14 M acetic acid/0.08 M sodium acetate). All other solvents and chemicals were purchased from Chempur (Piekary Śląskie, Poland).

### 3.2. Preparation of Microparticles

Drug-free and drug-loaded microparticles with chitosan or βGP/chitosan were prepared by using a Bűchi Mini Spray Dryer (Flawil, Switzerland) with a standard 0.5 mm nozzle. In order to select the optimal process parameters and obtain microparticles with desired properties, preliminary studies were conducted and the experimental parameters of the spray drying were set as follows: inlet temperature 155 °C–158 °C, outlet temperature 90 °C–92 °C, aspirator flow 37 m^3^/h, feed flow 1.5 mL/min, and spray flow 600 L/h. Microparticles were prepared using two drug/polymer mass ratios (1:4, 1:3) and different cross-linking agent/polymer ratios (1:3, 1:2, 1.5:2). Based on the preformulation experiments, concentration of chitosan solution 1.0% in 1.0% acetic acid (*w*/*v*) was selected for microparticles preparation.

For drug-free formulation, chitosan was solubilized in 1.0% (*w*/*v*) acetic acid solution and chilled in ice bath for 60 min. The appropriate amount of βGP (corresponding to polymer amount (*w*/*w*)) was next added to the cold chitosan base with continuous stirring. To ensure the high microbiological purity of microparticles, the feed solutions were prepared under aseptic conditions in a laminar flow cabinet Lamil Plus-13 with HEPA filters (Karstulan Metalli Oy, Karstula, Finland). The solutions were next sterilized using 0.22 μm membrane syringe filters (Millipore, Billerica, MA, USA) and subjected to spray drying. As CLO is sparingly soluble in water [58], to prepare drug-loaded microparticles, the drug was gradually suspended in a small amount of previously obtained chitosan or βGP/chitosan base, and then diluted with the remaining amount of the polymer base by transferring suspension to the baker with mechanical stirring (300 rpm, 1 h).

### 3.3. Morphology and Microparticle Size Analysis

Morphology and measurement of the microparticles size were performed using scanning electron microscopy SEM Hitachi SH 200 (Tokyo, Japan) after sputter-coating with gold (6.0 nm) in an argon atmosphere (Leica EM AC 2000, Wetzlar, Germany). The longest dimension from edge to edge of a single microparticle was measured for particles present in at least three areas of observation (×5000 magnification).

### 3.4. Drug Loading, Encapsulation Efficacy and Production Yield

CLO was extracted from accurately weighted amount of microparticles (10 mg) with a mixture of 1.0% acetic acid and methanol (1:4, *v*/*v*; 5 mL) under agitation at 150 rpm for 4 h in a water bath (25 °C) [59]. After filtration through 0.45 μm nylon Millipore filter (Millipore, Billerica, MA, USA), CLO concentration was determined by the HPLC method according to Hájková et al. [60] in the subsequent conditions: Zorbax Eclipse XDB–C18, 150 mm × 4.6 mm, 5 μm column (Agilent Technologies, Cary, NC, USA); mobile phase: methanol:phosphate buffer pH 7.4 (4:1, *v*/*v*), flow rate: 1.0 mL/min; UV detection 210 nm; retention time 5.4 min; the standard calibration curve was linear in the range from 1 to 100 μg/mL (*R*^2^ = 0.996).

### 3.5. Moisture Content Determination

In order to determine the percentage of moisture content, accurately weighted microparticles (50 mg) were placed in the aluminium pan of moisture analyser Radwag WPS 50SX (Radom, Poland), heated from 30 to 120 °C and the average of three measurements for each formulation was computed.

### 3.6. Differential Scanning Calorimetry

DSC measurements of CLO, chitosan, βGP, their physical binary mixtures in the ratio 1:1 (*w*/*w*) and a ternary mixture of CLO, βGP and chitosan with a ratio of 1:2:4 (*w*/*w*) (equaling the ratio of F2 formulation), placebo (P1 and P2) and drug-loaded microparticles (F1 and F2) were done on DSC-1 calorimeter (Mettler Toledo, Urdorf, Switzerland) equipped with an HSS-8 sensor. About 5 mg samples were placed in aluminum pans and hermetically sealed with lids. Analysis was performed under nitrogen atmosphere with a flow rate of 50 mL/min and heating rate of 10 °C/min. A simple heating program (from 0 °C to 310 °C) was used for all samples. Additionally, in order to assess changes in F2 microparticles under the influence of high temperature, a heating/cooling program cycle consisting of heating to 110 °C (kept at that temperature for 0 h or 1 h), cooling to 0 °C and heating again to 310 °C was used. Recorded thermograms were analyzed in the StarE program (version, Mettler Toledo, Urdorf, Switzerland).

### 3.7. Determination of the Mucoadhesive Properties

TA.XT.Plus Texture Analyser (Stable Microsystems, Godalming, UK) equipped with a 5 kg load cell, cylinder probe and the mucoadhesion measuring system A/MUC was applied for examination of mucoadhesive properties. The mucoadhesion of chitosan and βGP/chitosan microparticles were evaluated on porcine vaginal mucosa, which was attached to the upper probe with α-cyanoacrylate glue and lowered on the surface of microparticles with a constant speed of 0.5 mm/s [61]. Each formulation of microparticles (100 mg) was located on the platform below the texture analyser probe and moisturized with 100 μL of acetate buffer pH 4.5. The tests were conducted at 37 °C ± 0.5 °C. An acquisition rate of 200 points/s and a trigger force of 0.003 N were chosen for all measurements. After keeping contact for 100 s under an initial contact force 0.5 N, the factors were determined during preliminary studies, and the two surfaces were detached at a constant speed (0.1 mm/s). The maximum detachment force *F*_max_ (N) as a function of displacement was recorded directly from Texture Exponent 32 software (version 5.0, Stable Microsystems, Godalming, UK), whereas the work of mucoadhesion *W*_ad_ (expressed in μJ), was calculated from the area under the force vs. distance curve. Cellulose paper was used as a negative control.

### 3.8. Determination of the Residence Time

The in vitro residence time of microparticles was determined using a self-constructed apparatus according to Nakamura et al. [44] (by modifying USP Disintegration tester Electrolab ED-2L, Mumbai, India). Segments of porcine vaginal mucosa (2 cm long) were attached to the internal side of a beaker above the level of 500 mL acetic buffer pH 4.5 at 37 °C ± 0.5 °C. Microparticles (100 mg) were moisturized with acetic buffer (pH 4.5) and put in contact with the mucosal membrane. A plexiglass cylinder (weight 280 g, 6 cm diameter), vertically fixed to the apparatus, was allowed to move up and down to enable the complete immersion of microparticles in the buffer solution [61]. The time required for entire detachment of microparticles from the vaginal tissue was examined visually.

### 3.9. Swelling Index Test

Each formulation of microparticles was accurately weighted (50 mg) and placed in a Petri plate containing 2 mL acetic buffer (pH 4.5). At the predetermined time intervals (0.25, 0.5, 0.75, 1, 2, and 3 h), microparticles were removed, wiped off with filter paper, and reweighted. The swelling index was calculated by using the subsequent formula:

(1) $$SI=\frac{W_{2}-W_{1}}{W_{1}}$$

where *SI* was the swelling index, *W*_1_ was the initial weight of microparticles, and *W*_2_ was the weight of microparticles after the specific swelling time interval. Each test was performed in triplicate.

### 3.10. In Vitro Drug Release Studies

In vitro release of CLO from microparticles was assessed through natural cellulose membrane Cuprophan (Molecular Weight Cut-Off 10,000 Da, Medicell, London, UK) using an Enhancer cell (Agilent Technologies, Cary, NC, USA). The precisely weighted amount of each formulation of microparticles (referred to 10 mg of CLO) was added to 2 mL of release medium and the mixture was immediately located in the enhancer cell assembly making certain that no entrapped air was present at the membrane-drug carrier interface. Simple CLO dispersion in the release medium (1.0% (*w*/*v*)) was used as a control. A USP II dissolution tester (Agilent 708-DS, Agilent Technologies, Cary, NC, USA) equipped with mini paddles and mini vessels (250 mL) was applied to measure the CLO release from the drug reservoir [35]. The dissolution medium—acetic buffer pH 4.5 [57] with addition of 1.0% Cremophor EL—was maintained at 37 °C ± 0.5 °C and the rotation speed was 75 rpm. CLO solubility in the dissolution medium was 0.781 ± 0.091 mg/mL at 37 °C; in order to provide sink conditions, the volume of the medium was 100 mL. Samples (1 mL) were withdrawn at the predetermined time intervals (0.25, 0.5, 1, 2, 3, 4, 6, 8, 24 and 48 h), filtered through 0.45 μm cellulose acetate filters, diluted with mobile phase, and analyzed with the HPLC technique (as described in Section 3.4). Withdrawn samples were substituted with equal volumes of the fresh medium.

### 3.11. Cell Culture

VK2/E6E7 human vaginal mucosa cell line was maintained according to the attached procedure protocol in ker-SFM with 0.1 ng/mL human recombinant epidermal growth factor, 0.05 mg/mL bovine pituitary extract, 44.1 mg/mL calcium chloride (final concentration 0.4 mM), 50 U/mL penicillin and 50 μg/mL streptomycin. Cells were cultured in Costar flasks (Sigma Aldrich, Steinheim, Germany) and grown in a 5% CO_2_ atmosphere at 37 °C to attain subconfluence of 70%–80%. After discarding culture medium, confluent cells were rinsed with 0.25% (*w*/*v*) trypsin-0.03% (*w*/*v*) EDTA solution, counted in hemocytometer and seeded in 6-well or 24-well plates (Nunc, Thermo Scientific, Darmstadt, Germany) in 2 mL or 1 mL ker-SFM, respectively. Cultures were re-fed with 1–2 mL media per well every two to three days.

### 3.12. MTT Assay

Prior cytotoxicity studies, different amounts of freshly prepared sterile drug-loaded microparticles with unmodified and βGP cross-linked chitosan (F3, F5, F6) and placebo formulations (P3, P5, P6), which corresponded to a βGP:chitosan ratio of microparticles with CLO, were suspended in sterile acetate buffer pH 4.5 for 4 h under aseptic environment in a laminar flow cabinet Lamil Plus 13 (Karstulan Metalli Oy, Karstula, Finland). Next, the suitable amount of prepared samples were added to 24-well plates containing 1 × 10^5^ per well VK2/E6E7 confluent cells (to give final chitosan concentrations of 0.01 to 0.5 mg/mL) [62] and incubated for 4, 24 or 48 h at 37 °C in a 5% CO_2_ humidified atmosphere. The pH of the medium with samples varied in the range between 6.9 (for P3 and F3) to 7.32 (for P6 and F6 formulations). The cytotoxicity assay was performed according to the method of Carmichael et al. [63] with modifications using 3-(4,5-dimethylthiazol-2-yl)-2,5-diphenyltetrazolium bromide (MTT). Absorbance of converted dye in living cells was evaluated at a wavelength of 570 nm. Cell viability of VK2/E6E7 cells cultured in the presence of studied compounds was calculated as a percent of control cells. The control comprising cells not exposed to the chitosan formulations. After treatment of VK2/E6E7 cells with the samples, the ratio of survived to dead cells in tested and control cells was calculated for each concentration of unmodified or chitosan/βGP preparations.

### 3.13. Flow Cytometry Assessment of Annexin V-FITC/Propidium Iodide Binding

Apoptosis was determined by BD FACSCanto II flow cytometer (BD Biosciences Systems, San Jose, CA, USA) using an Apoptosis Detection Kit II according to the manufacturer’s instructions. Annexin V-FITC binds exclusively with phosphatidylserine, which is exposed at the cell surface during apoptosis [64], whereas propidium iodide (PI) selectively stains cells with a disrupted cell membrane and could be used to recognize late apoptotic and dead cells. The combination of annexin V-FITC with PI enables discrimination of viable cells that are unlabeled, apoptotic cells which are stained with annexin V-FITC, and necrotic cells—labeled with both annexin V-FITC and PI. Briefly, after 24 h incubation with the analyzed compounds, the cells were collected, washed twice with PBS, and resuspended in 100 μL binding buffer [65]. For each analysis, 10,000 VK2/E6E7 cells were counted. Cells cultured in a microparticle-free medium were used as controls. Forward scatter and side scatter signals were identified on a logarithmic scale histogram. FITC was detected in the FL1 channel (FL1 539, Threshold value 52). Results were analyzed with FACSDiva software (version 7.0, BD Bioscences Systems, San Jose, CA, USA).

### 3.14. Fluorescent Microscopy Assay

The morphology of VK2/E6E7 cells incubated for 24 h in the presence of unmodified chitosan or βGP cross-linked chitosan microparticles was observed by inverted fluorescence microscope Nikon Eclipse Ti-U (Nikon, Tokyo, Japan). Prior to the analysis, the cell suspension (250 μL) was stained with 10 μL of the dye mixture (10 μM acridine orange and 10 μM ethidium bromide), which was prepared in PBS. The control was cells incubated without analyzed compounds. The representative images were captured using a Nikon Digital Sight DS-Fi1c camera (Nikon, Tokyo, Japan) at a total of ×200 magnification.

### 3.15. Analysis of Mitochondrial Membrane Potential

Disruption of the mitochondrial membrane potential (MMP) was assessed using the lipophilic cationic probe 5,5′,6,6′-tetrachloro-1,1′,3,3′-tetraethylbenzimidazolcarbocyanine iodide (JC-1 MitoScreen kit; BD Biosciences, San Jose, CA, USA) as described previously [66]. Briefly, after 24 h incubation with the analyzed compounds, unfixed cells were washed and resuspended in PBS supplemented with JC-1. Cells were then incubated for 15 min at room temperature in the dark, washed, and resuspended in PBS for immediate BD FACSCanto II flow cytometry analysis. The percentage of cells with disrupted MMP was calculated in the FACSDiva software (version 7.0, BD Bioscences Systems, San Jose, CA, USA).

### 3.16. Statistical Analysis

Quantitative variables were expressed as the mean ± standard deviation and (or) the median. A statistical analysis was accomplished using nonparametric methods: the Kruskal-Wallis and Mann-Whitney-U test with using the Statistica 12.5 software (StatSoft, Kraków, Poland). Differences between groups were reflected to be significant at *p* < 0.05.

## 4. Conclusions

To our best knowledge, this work demonstrated for the first time the preparation of chitosan microparticulate delivery carriers by spray drying while using βGP as cross-linking agent. A particular effort was made to evaluate the impact of βGP, applied in different ratios, on chitosan microparticles’ characteristics. Unmodified chitosan microparticles had a rough surface, whereas smoother and more intact microparticles F5 and F6 (with βGP:chitosan ratio 1:2 and 1.5:2 (*w*/*w*), respectively) were observed. The microparticles’ size and drug loading were found to decrease with raising the amount of βGP. Microparticles with βGP/chitosan ratio 1:2 (*w*/*w*) exhibited the highest loading efficacy. In addition, the amount of ion cross-linker had a remarkable prolonged effect on the drug release behavior from βGP/chitosan microparticulate dosage forms. All investigated formulations exhibited beneficial mucoadhesive properties and displayed prolonged residence time to porcine vaginal mucosa. A drop in work of adhesion observed for βGP/chitosan microparticulate might be a result of more intact particle surface and lower water content, leading to formation of a less cohesive structure with mucosal tissue.

The data from the cytotoxicity studies indicated that chitosan and βGP/chitosan microparticles affected cell viability and accelerated the rate of apoptosis in human vaginal epithelial cells in a dose-dependent manner. The cytotoxic effect of unmodified and βGP cross-linked chitosan was hardly affected by the presence of drugs. Notably, mitochondrial integrity measured by JC-1 assay appeared to play a substantial role in apoptosis induced by chitosan microparticles in VK2/E6E7 cells. The findings from the cytotoxicity studies added novel insight into the potential safety profile of spray-dried chitosan microparticles and could help to better understand the toxicity of chitosan microparticulate delivery systems.

## Figures and Tables

**Table 1:** Characteristics of chitosan (CS) and βGP/CS microparticles with clotrimazole (CLO).

Formu-lation	βGP:CS Ratio (*w*/*w*)	CLO:CS Ratio (*w*/*w*)	Production Yield (%) ^a^ *	Encapsulation Efficacy (%) ^b^ *	CLO Loading (%) ^c^ *	Diameter Range (Mean Diameter *) (μm)	Moisture Content (%) *	Residence Time (min) *
*1.0% (w*/*v) chitosan solution*								
F1	-	1:4	75.4 ± 1.3	59.5 ± 3.1	11.2 ± 2.8	*n.t.*	14.3 ± 3.5	*n.t.*
F2	1:2	1:4	78.0 ± 1.6	58.2 ± 3.9	8.3 ± 2.7	*n.t.*	8.9 ± 2.6	*n.t.*
F3	-	1:3	73.5 ± 0.9	67.2 ± 9.1	16.8 ± 4.1	1.52–5.28 (3.39 ± 1.87)	10.4 ± 2.9	60 ± 2
F4	1:3	1:3	78.8 ± 1.2	59.6 ± 1.0	11.9 ± 2.7	1.49–6.33 (3.96 ± 2.45)	7.6 ± 2.2	116 ± 4
F5	1:2	1:3	79.5 ± 1.1	73.2 ± 9.0	13.3 ± 3.2	1.23–5.25 (3.12 ± 2.13)	6.5 ± 2.4	132 ± 5
F6	1.5:2	1:3	80.6 ± 1.9	68.4 ± 11.3	10.9 ± 2.1	0.93–3.93 (2.54 ± 1.59)	7.9 ± 2.7	154 ± 5

^a^ the weight of obtained microparticles/overall weight of components in feed solution ×100; ^b^ actual CLO content in microparticles/theoretical CLO content ×100; ^c^ actual CLO content in microparticles/examined amount of microparticles ×100; * mean ± S.D; *n* = 3; *n.t.*—not tested.

**Table 2:** Abbreviations used in the in vitro cytotoxicity evaluation.

Abbreviation	Description	
K0		non treated cells
K1	Cells treated with:	acetic buffer pH 4.5
K2		aqueous solution of βGP *
K3		aqueous solution of βGP **
P3/F3		unmodified chitosan placebo/drug-loaded microparticles
P5/F5		βGP:chitosan (1:2) placebo/drug-loaded microparticles
P6/F6		βGP:chitosan (1.5:2) placebo/drug-loaded microparticles

The amount of βGP (*w*/*w*) added corresponded with those added to chitosan microparticles (*) P5, F5; (**) P6, F6.
